# The Association between Nitric Oxide Pathway, Blood Pressure Abnormalities, and Cardiovascular Risk Profile in Pediatric Chronic Kidney Disease

**DOI:** 10.3390/ijms20215301

**Published:** 2019-10-24

**Authors:** Chien-Ning Hsu, Pei-Chen Lu, Mao-Hung Lo, I-Chun Lin, You-Lin Tain

**Affiliations:** 1 Department of Pharmacy, Kaohsiung Chang Gung Memorial Hospital and College of Medicine, Chang Gung University, Kaohsiung 833, Taiwan; chien_ning_hsu@hotmail.com; 2 School of Pharmacy, Kaohsiung Medical University, Kaohsiung 807, Taiwan; 3 Department of Pediatrics, Kaohsiung Chang Gung Memorial Hospital and College of Medicine, Chang Gung University, Kaohsiung 833, Taiwan; alexiellu@gmail.com (P.-C.L.); trentlo@cgmh.org.tw (M.-H.L.); uc22@cgmh.org.tw (I.-C.L.)

**Keywords:** ambulatory blood pressure monitoring, arterial stiffness, asymmetric dimethylarginine, cardiovascular disease, children, chronic kidney disease, hypertension, left ventricular mass index, nitric oxide, pulse wave velocity

## Abstract

Cardiovascular disease (CVD) is common in chronic kidney disease (CKD), while major CV events are rare in young CKD patients. In addition to nitric oxide (NO)-related biomarkers, several surrogate markers have been assessed to stratify CV risk in youth with CKD, including 24-h ambulatory blood pressure monitoring (ABPM), carotid artery intima-media thickness (cIMT), pulse wave velocity (PWV), ABPM-derived arterial stiffness index (AASI), flow-mediated dilatation (FMD), and left ventricular mass index (LVMI). The aim of this study was to identify subclinical CVD through the analysis of indices of CV risk in children and adolescents with CKD. Between 2016 and 2018, the prospective observational study enrolled 125 patients aged 3 to 18 years with G1–G4 CKD stages. Close to two-thirds of young patients with CKD exhibited blood pressure (BP) abnormalities on ABPM. CKD children with abnormal office BP showed lower plasma arginine levels and arginine-to-asymmetric dimethylarginine (ADMA) ratio, but higher ratios of ADMA-to-symmetric dimethylarginine (SDMA) and citrulline-to-arginine. High PWV and AASI, indices of arterial stiffness, both strongly correlated with high BP load. Additionally, LV mass and LVMI exhibited strong correlations with high BP load. Using an adjusted regression model, we observed the citrulline-to-arginine ratio was associated with 24-h systolic and diastolic BP, systolic blood pressure (SBP) load, and diastolic blood pressure (DBP) load. Early assessments of NO-related parameters, BP load abnormalities, arterial stiffness indices, and LV mass will aid in early preventative care toward decreasing CV risk later in life for children and adolescents with CKD.

## 1. Introduction

Non-communicable diseases (NCDs) present the greatest public health challenges of the 21st century and account for two-thirds of all global deaths [1]. Cardiovascular disease (CVD) alone makes up approximately 50% of all NCD deaths. In general, major CV events tend to occur in adults and are rare in children. However, the stiffening of the arteries can begin in early life. Subclinical CVD presenting in children is not easily detectable by conventional methods [2]. Children with chronic kidney disease (CKD) are at high risk of developing CVD [3]. By the time CKD children require dialysis or kidney transplantation, their CV mortality is 30-fold higher than the general population [4], indicating the need for early identification and prevention.

Surrogate markers have been required to stratify risk for CVD in children. Some biomarkers could evolve to act as surrogates including biomarkers, structural markers, or functional markers. Nitric oxide (NO), a potent vasodilator, plays a relevant role in the regulation of blood pressure (BP) and renal physiology [5]. Emerging evidence supports that NO deficiency is involved in the pathogenesis of hypertension and CKD [6,7,8]. Arginine is a substrate for nitric oxide synthase (NOS) to generate NO and citrulline. Because arginine has multiple metabolic fates and the kidney can use citrulline to make arginine [9,10], there are conflicting findings in the literature according to its plasma level as a biomarker for CKD and hypertension [11,12]. Asymmetric dimethylarginine (ADMA) and symmetric dimethylarginine (SDMA) are endogenous inhibitors of NOS [13,14]. In adult CKD patients, ADMA and SDMA are independent risk markers for mortality and CVD [15]. Unfortunately, very limited information is available on the simultaneous analysis of these components in the NO pathway to explore their impact on CVD in pediatric CKD [16,17]. 

Several structural and functional markers have been proposed to stratify the cardiovascular risk in CKD patients, including left ventricular mass [2], 24-h ambulatory blood pressure monitoring (ABPM) [17,18], carotid artery intima-media thickness (cIMT) [19], arterial stiffness indices (e.g., pulse wave velocity [PWV] and ABPM-derived arterial stiffness index [AASI]) [20], and flow-mediated dilatation (FMD) [21]. However, the understanding of these markers in pediatric CKD remains limited. Therefore, the aim of this study was to identify the associations between NO-related biomarkers, BP load, and structural and functional markers in children and adolescents with early-stage CKD.

## 2. Results

We enrolled a total of 125 children and adolescents with CKD stage G1–G4 in this study. The clinical characteristics of the study participants are presented in Table 1, overall and by office BP. The median age was 9.6 years, 58% were male. Our study cohort had a median estimated glomerular filtration rate (eGFR) of 107 mL/min/1.73 m^2^, which indicates that most participants had CKD in the early stage. Congenital anomalies of the kidney and urinary tract (CAKUT) were the underlying kidney disease in 64% of patients. The diagnosis of office BP depends on age, gender, and height. Systolic BP (SBP) and/or diastolic BP (DBP) in the 95th percentile or greater was considered abnormal (i.e., hypertension). The adult cut-off was applied at persons aged 16–18 years [22]. Of the 125 patients, 48 cases (38%) were found to have abnormal office BP. As shown in Table 1, children with abnormal office BP had heavier proteinuria, higher plasma levels of total cholesterol, low-density lipoprotein (LDL), and uric acid compared to those with normal office BP.

The plasma levels of NO-related parameters and their combined ratios stratified by the office BP are illustrated in Table 2. The plasma arginine level and arginine-to-ADMA ratio were lower in children with abnormal office BP than in those with normal office BP. Conversely, the ADMA-to-SDMA and citrulline-to-arginine ratios were higher in the abnormal vs. normal office BP group. Plasma levels of citrulline, ADMA, and SDMA, however, were not different between the two groups. We also analyzed the differences of NO-related parameters between patients with CKD stage G2–G4 vs. CKD stage 1. Patients with CKD stage G2–G4 had a higher citrulline level (38 (IQR 29.2–58.3) vs. 30.6 (IQR 24.2–39.8), *p* = 0.021) while a lower ADMA-to-SDMA ratio (1.37 (IQR 1.03–1.83) vs. 1.74 (IQR 1.55–2.05), *p* = 0.042) than those with CKD stage G1. No significant differences in arginine, ADMA, SDMA, arginine-to-ADMA ratio, and citrulline-to-arginine ratio were observed between CKD stage G2–G4 and the CKD stage 1 group.

A complete analysis of 24-h ABPM study was feasible in 76 patients aged 6–18 years with CKD (Table 3). After ABPM, we identified: 11 (15%) with systolic BP (SBP) or diastolic BP (DBP) load > 95th percentile at 24-h, 12 (16%) as having daytime hypertension, and 18 patients (24%) as having nighttime hypertension. Additionally, 37 patients (49%) had a BP load ≥ 25% and 32 patients (42%) exhibited nocturnal non-dipping. In the present study, a total of 48 (63%) of CKD children were identified with at least one BP load abnormality on 24-h ABPM. Patients with CKD stage G2–G4 had a higher 24-h SBP (*p* < 0.001), 24-h DBP (*p* = 0.043), SBP load (*p* = 0.004), and DBP load (*p* = 0.039) than those with CKD stage G1. The rate of patients with abnormal 24-h, daytime BP, nighttime BP, and BP load was higher among patients with CKD stage G2–G4 than those with CKD stage 1. The PWV, an arterial stiffness index, and LV mass were higher in children with CKD stage G2–G4 compared to those with G1. However, cIMT, FMD, AASI, and left ventricular mass index (LVMI) were not different between CKD children with stage G1 and G2–G4. Also, we analyzed the differences in ABPM profile and cardiovascular indices between CKD patients with CAKUT and with non-CAKUT. Nevertheless, we found all cardiovascular indices were comparable between these two groups (All *p* > 0.05).

As shown in Table 4, we further analyzed indices of cardiovascular structure and function in CKD children by ABPM. We observed that PWV was higher in children with abnormal 24-h, daytime, and nighttime BP, increased BP load, and nocturnal non-dipping than those with normal ABPM profile. Additionally, CKD children with abnormal 24-h, daytime, and nighttime BP in the ABPM profile had a higher AASI compared to those with a normal ABPM profile. Like PWV, LVMI was higher in CKD children with abnormal vs. normal ABPM profile.

Associations between NO-related biomarkers and BP load were further examined in a multivariate linear regression model adjusted for age, sex, and creatinine (Table 5). To specify the exact role of each NO-related biomarker in BP load, a multivariate linear regression model using the stepwise selection was applied for age, sex, and creatinine with some other NO-related biomarkers. In the best predictive model (*r* = 0.613, *p* < 0.001), the citrulline-to-arginine ratio was significantly associated with 24-h systolic BP (*p* = 0.006) controlling for age and creatinine. Also, the citrulline-to-arginine ratio was associated with 24-h diastolic BP (*p* < 0.001) controlling for creatinine. We also found that both citrulline-to-arginine ratio and citrulline had significant associations with SBP load (*p* = 0.001 and 0.032, respectively) controlling for creatinine in a predictive model applied for the above-mentioned factors (*r* = 0.691, *p* < 0.001). Additionally, a negative association was found for citrulline-to-arginine ratio and DBP load in the adjusted regression model (*r* = 0.642, *p* < 0.001).

Correlation analysis of NO-related biomarkers and cardiovascular surrogate markers is detailed in Table 6. We found that arginine was positively correlated with cIMT (*r* = 0.346, *p* = 0.003) and PWV (*r* = 0.258, *p* = 0.029). Furthermore, citrulline-to-arginine ratio had a negatively correlation with cIMT (*r* = −0.342, *p* = 0.003), LV mass (*r* = −0.267, *p* = 0.024), and LVMI (*r* = −0.302, *p* = 0.011). However, citrulline, ADMA, SDMA, arginine-to-ADMA-ratio, and ADMA-to-SDMA ratio were not correlated with any of the cardiovascular surrogate markers. A multivariate linear regression model was further analyzed to control for the effects of covariates (e.g., age, sex, creatinine) on cardiovascular surrogate markers. When the covariates were taken into account, only arginine had a significant association with cIMT (*Beta* = 0.384, *p* = 0.002) in a multivariable regression model applied for age, sex, creatinine, and NO-related biomarkers (*r* = 0.384, *p* = 0.002). However, other relationships were no longer significant.

## 3. Discussion

This study provides insight into a link between NO-related biomarkers, structural and functional markers, and cardiovascular risk in children and adolescents with early-stage CKD. Our major findings are as follows: (1) approximately two-thirds of children and adolescents with CKD stage G1–G4 had BP abnormalities on ABPM; (2) plasma arginine level and arginine-to-ADMA ratio were lower, whereas ADMA-to-SDMA and citrulline-to-arginine ratios were higher in CKD children with abnormal office BP; (3) PWV and LV mass were increased in the case of CKD stage G2–G4 compared to those with stage G1; (4) PWV and LVMI was higher in CKD children with abnormal vs. normal ABPM profile; (5) PWV, LV mass, and LVMI exhibited stronger correlations with elevated BP load than other cardiovascular surrogate markers; and (6) the citrulline-to-arginine ratio was associated with 24-h SBP, 24-h DBP, SBP load, and DBP load in the adjusted regression model.

In line with previous studies in pediatric CKD [3,16,23,24], our study documented an extremely high prevalence of subclinical CVD in CKD children characterized by abnormalities on ABPM, LV mass, and arterial stiffness. In the current study, up to 63% of children with mild to moderate CKD displayed BP abnormalities, despite only 38% of CKD children having abnormal office BP. Our data support the concept that the identification of BP abnormalities not evident in casual BP readings in pediatric CKD has significantly improved with the use of ABPM [25]. We observed several well-known CVD risk factors such as cholesterol, LDL, proteinuria, and uric acid were correlated to CKD children with abnormal office BP. Among them, uric acid showed a strong positive correlation with elevated BP load. Additionally, we observed that PWV and AASI, indices of arterial stiffness, both are strongly correlated with uric acid. These findings are supported by a previous study of adult patients with CKD, in whom a worse arterial stiffness is related to hyperuricemia-induced hypertension [26]. Since uric acid is considered not only a CVD risk factor but also a potential therapeutic target in CKD [27], more studies are needed to clarify whether early targeting on uric acid can prevent the development of CVD in children with CKD, even in an early stage.

Although most evidence indicates NO deficiency in CKD and hypertension [6,7,8], some studies reported that NO synthesis was increased in patients with CKD [28]. In the current study, CKD children with hypertension exhibited a low plasma arginine level, low arginine-to-ADMA ratio, and high ADMA-to-SDMA ratio. As arginine is the substrate for NOS and arginine-to-ADMA ratio is referred to as an index of NO bioavailability [29], our results suggest NO deficiency is related to the development of hypertension in children and adolescents with early-stage CKD. In the present study, FMD was assessed to evaluate endothelial dysfunction, a condition that entails less local availability of NO [21]. However, we did not observe the associations between FMD and NO-related biomarkers, BP load, and other CV surrogate markers. It is possible that the small number of patients included may not reach sufficient power to determine small differences in early stage of CKD. Although a previous report demonstrated that plasma ADMA and SDMA levels positively correlate with BP load in children with CKD [17], such findings are not supported by the present data. Unlike ADMA, SDMA is excreted by the kidneys only. A high ADMA-to-SDMA ratio could be suggestive of reduced ADMA excretion or metabolism [30]. Thus, ADMA-to-SDMA ratio may provide information about the dimethylarginine dimethylaminohydrolase (DDAH) activity because only ADMA is metabolized by the enzyme [14]. We observed that hypertension is related to a higher ADMA-to-SDMA ratio in CKD children. In agreement with a previous study showing that increased DDAH activity can reduce ADMA and prevent hypertension in young spontaneously hypertensive rats [31], our data suggest decreased DDAH activity might be involved in the development of hypertension. When the covariates were taken into account, the citrulline-to-arginine ratio was the only NO-related marker, which could well explain BP load in the adjusted regression model. In patients with CKD, a decreased renal uptake of citrulline and its conversion to arginine can result in a high plasma citrulline-to-arginine ratio. We observed that citrulline and citrulline-to-arginine ratio both were negatively associated with SBP load. Given that CKD children with a high BP load had a low citrulline-to-arginine ratio, it is possible a high conversion rate of citrulline to arginine generated NO as a compensatory response in the pre-hypertensive stage. In adult CKD patients, higher plasma ADMA and SDMA levels were reported in patients with more advanced CKD [32]. These findings are not supported by the results of the present analyses, which demonstrate that almost all NO-related parameters did not differ in CKD children with stage G2–G4 vs. stage G1. Thus, the roles of NO-related biomarkers in CKD progression in children await further clarification. 

As expected, the severity of CKD is associated with certain markers of adverse CV health, like PWV and LV mass in the current study. Although PWV has been used in children, age-specific reference ranges are still unavailable [2]. In the present study, PWV was increased in children with abnormal 24-h, daytime, and nighttime BP, increased BP load, and nocturnal non-dipping. Additionally, PWV was strongly correlated with 24-h SBP, 24-h DBP, SBP load, and DBP load. Like PWV, the AASI is an indirect arterial stiffness index [33]. Our results demonstrated that high AASI is related to abnormal 24-h, daytime, and nighttime BP in the ABPM profile in children with CKD. The present results are in agreement with the previous study in that arterial stiffness worsens in CKD patients with masked hypertension [34]. In addition to PWV and AASI, cIMT might also have a smaller role in contributing to BP load. We found a weaker correlation between cIMT and high BP load than indices of arterial stiffness. It is possible that arterial stiffness enables evaluation of arterial dysfunction, which may precede structural vascular remodeling evaluated by cIMT. Left ventricular hypertrophy (LVH), an index of target organ damage, poses an increased risk for CVD in patients with CKD [35]. In keeping with previous studies showing that ABPM correlates with LV hypertrophy in CKD children [18,36], we found LV mass and LVMI both strongly correlated with systolic and diastolic BP load on ABPM.

Our study was not without its limitations. First, additional assessments of the above-mentioned CV markers with longer follow-ups are essential in view of the long-term nature of childhood CKD. Second, there are no reference values established for PWV, AASI, cIMT, and LVMI to define a cut-off point between healthy and CKD children [2]. Third, there might be an ethnic difference as we used BP nomograms and ABPM reference values from studies in white people [37,38]. Fourth, two new hypertension guidelines in children and adolescents have been released recently [22,39] and result in discrepancies on BP nomograms, the definition of BP categories based on percentiles, as well as the adult cut-off age. Thus, a plan of action needs to evaluate the impact of different guidelines that have introduced redefinitions of BP categories on the above-mentioned CV markers and CV outcomes. Last, the small number of patients included might not reveal a true relationship. Larger numbers of CKD children recruited via multi-center cohorts may be warranted.

## 4. Materials and Methods

### 4.1. Study Population

The prospective cohort study was approved by the Institution Review Board and Ethics Committee of Chang Gung Medical Foundation, Taoyuan, Taiwan (Permit number: 201601181A3; approval date: 30 December 2016) and adherent to the principle of the 1964 Declaration of Helsinki and its later amendments. All participants gave written informed consent. The present study includes 125 participants who contributed at least one visit to a pediatric nephrology clinic in a Taiwan medical center between December 2016 and October 2018, while they were between 3 to 18 years of age with CKD. CKD is defined as kidney damage or decreased kidney function over at least three months [40]. Kidney damage refers to structural abnormalities or functional abnormalities, whether established via renal biopsy or imaging studies, or inferred from markers such as urinary sediment abnormalities or proteinuria. Renal function was determined by the estimated glomerular filtration rate (eGFR) using the Schwartz formula according to body height and serum creatinine level [41]. Individuals who were excluded from the study were those who: (1) had a documented pregnancy; (2) had a congenital heart disease; (3) had an eGFR < 15 mL/min/1.73 m^2^, or were on dialysis maintenance or had ever received a renal transplantation; or (4) were unable to follow-up protocol or cooperate with the assessment. All participants were categorized to eGFR categories: G1 (eGFR ≥ 90 mL/min/1.73 m^2^), G2 (eGFR 60–89 mL/min/1.73 m^2^), G3 (eGFR 30–59 mL/min/1.73 m^2^), or G4 (eGFR 15–30 mL/min/1.73 m^2^). The analysis was restricted to children with a baseline eGFR > 15ml/min/1.73m^2^, who measured renal and cardiovascular parameters described in the following section. The causes of kidney disease were divided into two categories: Congenital anomalies of the kidney and urinary tract (CAKUT), or non-CAKUT. CAKUT structural anomalies range from renal agenesis, kidney hypoplasia, kidney dysplasia, multi-cystic kidney dysplasia, horseshoe kidney, duplex collecting system, posterior urethral valves, and ureter abnormalities [42].

### 4.2. Biochemical Analysis

Fasting plasma specimens, spot urine, and fecal samples were aliquoted and stored at –80 °C until analysis. Blood urea nitrogen, creatinine, uric acid, glucose, total cholesterol, LDL, triglyceride, sodium, potassium, calcium, phosphate, hemoglobin, and urine total protein-to-creatinine ratio were measured by the hospital central laboratory. We directed the families to prevent their children from having an excessive intake of foods rich with arginine and citrulline (e.g., peanut, gelatin, and watermelon) for 1 week before blood and urine sampling.

### 4.3. Office BP and 24-h ABPM

BP measurements were taken at the clinic visit. The participant rested quietly for 5 min in a seated position. Three BP recordings were obtained 30 s apart by experienced specialist nurses using a standard mercury sphygmomanometer (Philips MP5, Boeblingen, Germany). A cuff with a bladder sized by a 1:2 width to length ratio according to the size of the arm was chosen. The mean value was used as the participant’s office BP for analysis. The 24-h ABPM data were collected for subjects aged 6–18 years using an Oscar II monitoring device (SunTech Medical, Morrisville, NC, USA) handled by a trained specialist nurse as previously reported [42]. Briefly, the ABPM was set to record the BP and pulse rate at 20-min intervals over 24-h. The subjects and their parents were asked to keep a diary of sleeping and waking times, as well as activities that may influence BP measurements, including exercise and stressful situations. Only measurements with a systolic BP of 50–200 mmHg, a diastolic BP of 30–100 mmHg, and a heart rate of 30–200 beats per min were accepted as valid and included in analysis. If more than 25% of an individual’s recordings were outside these valid ranges, then that individual was excluded from further analysis.

An abnormal ABPM profile was determined based on: (1) awake, asleep, systolic, or diastolic BP loads exceeding the 95th percentile based on gender and height using ABPM reference data [38]; (2) awake, asleep, systolic or diastolic BP load of 25% or greater; and (3) asleep decrease of BP load by less than 10% compared with average awake BP load. Additionally, we calculated the AASI as one minus the slope of the DBP reading over the SBP reading [34].

### 4.4. Carotid Ultrasonography and Echocardiography

The ultrasound studies were performed by two experienced pediatricians (P.-C.L. and I.-C.L.) as previously reported [16]. Patients were in the supine position for at least 10-min in a quiet room prior to examination. The patient’s chin was hyperextended and turned toward the contralateral side at about a 45-degree angle from the midline. The mid common carotid artery was imaged using a 5- to 12-MHz linear array transducer (Aloka Co., Tokyo, Japan). We used the distance between the leading edges of the luminal-intimal interface and the medial-adventitial interface for the measurement of cIMT. The cIMT was measured during end-diastole as determined by the R wave on the electrocardiogram. These images were obtained by the ProSound α7 ultrasound coupled to computer-assisted analysis software (ProSound α7, e-TRACKING system; Aloka Co., Tokyo, Japan). PWV was determined by echo-tracking methods (e-TRACKING system; Aloka Co., Tokyo, Japan). Endothelial function was analyzed by measuring FMD, defined as the maximal percentage change in right brachial arterial diameter in response to reactive hyperemia induced by inflating a BP cuff to >200 mmHg around the forearm for 5 min. Brachial artery diameter was measured in longitudinal section with a UST-5412 linear probe and a ProSound a7 ultrasound system coupled to computer-assisted analysis software, as previously described [16]. Each study included a resting scan and reactive hyperemia scan recorded 30–60 s after the cuff pressure was released, which occluded arterial blood flow for 5 min. Additionally, all participants underwent a transthoracic echocardiographic examination with commercially available machines (Philips IE33 system, Philips, Bothell, WA, USA). The left ventricular mass (LVM) was calculated using images obtained in the parasternal long-axis or short-axis view of the left ventricle by M-mode echocardiography. The LVM index (LVMI) was obtained by indexing LVM to height^2.7^ [43].

### 4.5. HPLC Analysis

Plasma levels of citrulline, arginine, ADMA, and SDMA were determined using high-performance liquid chromatography (HPLC: HP series 1100, Agilent Technologies, Inc, Santa Clara, CA, USA). O-phthaldialdehyde 3-mercaptopropionic acid (OPA-3MPA) was used for the fluorescent derivatization [44]. The standards contained arginine and citrulline at 1–100 μmol/L. For ADMA and SDMA, the standards were set at 0.5–5 μmol/L.

### 4.6. Statistical Analysis

Data were expressed as median (25th percentile–75th percentile) or *n* (%), as appropriate. Group differences were analyzed by Mann–Whitney U-test or Chi-square test, as appropriate. The associations between variables were assessed using Spearman’s rank correlations. A linear regression model was performed followed by the stepwise multivariable analyses integrating relevant parameters to explain BP load. A value of *p* < 0.05 was considered statistically significant. Statistical Package for the Social Sciences (SPSS) software 14.0 (Chicago, IL, USA) was used. 

## 5. Conclusions

In conclusion, a significant proportion of children with CKD, even in early-stage, exhibit BP abnormalities and an adverse CV risk profile. Although the mechanism underlying the NO pathway and CVD in CKD children has not yet been completely clarified, our results cast a new light on the link between NO pathway, BP load, and arterial stiffness in children with CKD. Early detection of NO-related biomarkers and structural and functional surrogate markers may assist in advancing knowledge of CVD and initiating treatment at an early stage with respect to improving CV outcome in at-risk children with CKD.

## Figures and Tables

**Table 1:** Patient characteristics in children and adolescents with CKD.

	All Patients	Office BP	
		Normal	Abnormal
Characteristic	*N* = 125	*N* = 77	*N* = 48
Age, years	9.6 (5.3–14.4)	10 (6.4–14.2)	8.7 (4.8–15.7)
Male	72 (57.6%)	41 (53.2%)	31 (64.6%)
CKD staging			
Stage G1	91 (72.8%)	59 (76.6%)	32 (66.7%)
Stage G2	22 (17.6%)	12 (15.6%)	10 (20.8%)
Stage G3	10 (8%)	6 (7.8%)	4 (8.3%)
Stage G4	2 (1.6%)	0 (0%)	2 (4.2%)
CAKUT	80 (64%)	51 (66.2%)	29 (60.4%)
Body height, percentile	50 (20–75)	50 (25–75)	25 (15–75)
Body weight, percentile	50 (15–85)	50 (15–75)	63 (15–85)
Systolic blood pressure, mmHg	109 (100–120)	103 (98–114)	119 (109–136) *
Diastolic blood pressure, mmHg	69 (63–77)	64 (60–71)	77 (68–83) *
Body mass index, kg·m^−2^	17.6 (15.6–20.7)	17.1 (15.2–20.3)	18 (16.1–22.3)
Blood urea nitrogen, mg/dL	12 (10–15)	12 (10–14)	13 (10–17)
Creatinine, mg/dL	0.5 (0.42–0.69)	0.51 (0.44–0.67)	0.5 (0.37–0.7)
eGFR, mL/min/1.73 m^2^	107 (87–125)	110 (91–125)	102 (83–126)
Urine total protein-to-creatinine ratio, mg/g	69 (40–283)	61 (38–169)	128 (43–1424) *
Hemoglobin, g/dL	13.4 (12.6–14.2)	13.4 (12.6–14.2)	13.4 (12.4–14.6)
Hematocrit, %	39.7 (37.3–41.8)	39.6 (37.3–41.8)	40 (37.3–42.6)
Total cholesterol, mg/dL	165 (146–190)	160 (140–179)	177 (154–202) *
LDL, mg/dL	87 (71–105)	81 (65–100)	94 (78–128) *
Triglyceride, mg/dL	67 (51–103)	62 (50–92)	72 (51–142)
Fasting glucose, mg/dL	87 (83–92)	87 (83–91)	87 (83–94)
Uric acid, mg/dL	5.3 (4.1–6.3)	4.9 (4–6.1)	5.8 (5–7) *
Sodium, mEq/L	141 (140–142)	141 (140–142)	141 (140–142)
Potassium, mEq/L	4.4 (4.2–4.6)	4.4 (4.2–4.5)	4.4 (4.1–4.6)
Calcium, mg/dL	9.7 (9.3–9.9)	9.7 (9.4–9.9)	9.6 (9.1–10)
Phosphate, mg/dL	4.9 (4.5–5.3)	4.9 (4.5–5.3)	5 (4.4–5.4)
Ca × P product, mg^2^/dL^2^	47.5 (41.7–51.8)	47 (41.8–51.8)	47.6 (41.4–51.8)

Data are given as median (25th percentile–75th percentile) or *n* (%) as appropriate. * *p* < 0.05 children (age < 12 years old) vs. adolescent (age > 12 years old) by the Chi-square test or Mann–Whitney U-test. BP = blood pressure; CAKUT = Congenital anomalies of the kidney and urinary tract; eGFR = estimated glomerular filtration rate; LDL = low-density lipoprotein.

**Table 2:** Plasma levels of NO pathway components in children and adolescents with CKD.

Office BP	Normal	Abnormal
	*N* = 74	*N* = 47
Citrulline, μM	36 (25.7–46.1)	37.6 (27.5–47.2)
Arginine, μM	92.2 (71–109)	78.5 (57.5–98.9) *
ADMA, μM	1.05 (0.7–1.33)	1.1 (0.8–1.3)
SDMA, μM	0.7 (0.6–0.93)	0.6 (0.5–0.8)
Arginine-to-ADMA ratio, μM/μM	89.5 (54.2–141.4)	74.8 (57.2–93.3) *
ADMA-to-SDMA ratio, μM/μM	1.5 (1–1.91)	1.71 (1.43–2.33) *
Citrulline-to-Arginine ratio, μM/μM	0.4 (0.3–0.58)	0.51 (0.39–0.68) *

Data are given as median (25th percentile–75th percentile). * *p* < 0.05 by the Mann-Whitney U-test. NO = nitric oxide; SCFAs = short chain fatty acids; ADMA = asymmetric dimethylarginine; SDMA = symmetric dimethylarginine.

**Table 3:** Cardiovascular characteristics in children and adolescents with CKD.

CKD Stage	G1	G2–G4	Total
24-h ABPM	*N* = 50	*N* = 26	*N* = 76
24-h systolic BP	109 (101–116)	120 (112–135) *	112 (103–121)
24-h diastolic BP	63 (58–66)	65 (61–72) *	64 (59–68)
SBP load	2.5 (0–15)	21 (2.8–45.3) *	4 (2–27.8)
DBP load	2 (0–7.2)	5 (0–16.3) *	2 (0–8)
Abnormal ABPM profile (with any of the following abnormalities)	26 (52%)	22 (85%) *	48 (63%)
Average 24-h BP > 95th percentile	4 (8%)	7 (27%) *	11 (15%)
Average daytime BP > 95th percentile	4 (8%)	8 (31%) *	12 (16%)
Average nighttime > 95th percentile	8 (16%)	10 (39%) *	18 (24%)
BP load ≥ 25%	17 (34%)	20 (77%) *	37 (49%)
Nocturnal decrease of BP < 10%	19 (38%)	13 (50%)	32 (42%)
cIMT, mm	0.3 (0.3–0.4)	0.3 (0.3–0.4)	0.3 (0.3–0.4)
PWV, m/s	3.8 (3.4–4.2)	4 (3.6–4.8) *	3.85 (3.53–4.38)
FMD, %	8.3 (3.4–12.9)	4.6 (2.6–15.4)	7.8 (3.2–12.9)
AASI	0.37 (0.22–0.43)	0.39 (0.33–0.48)	0.36 (0.24–0.44)
LV mass, g	87.5 (66.5–115)	123 (73.7–159) *	94.9 (66.5–127)
LVMI, g/m^2.7^	30.7 (25.6–37.2)	31 (28.6–39)	30.9 (26.1–38.2)

Data are given as median (25th percentile–75th percentile) or *n* (%) as appropriate. * *p* < 0.05 by the Chi-square test. ABPM = 24-h ambulatory blood pressure monitoring. cIMT = carotid artery intima-media thickness. PWV = pulse wave velocity. FMD = flow-mediated dilatation. AASI = ABPM-derived arterial stiffness index. LVMI = left ventricular mass index.

**Table 4:** Cardiovascular parameters vs. ABPM profile in children and adolescents with CKD.

BP	cIMT, mm	PWV, m/s	FMD, %	AASI	LVMI, g/m^2.7^
24-h BP					
Abnormal	0.4 (0.3–0.4)	4.1 (4–5) *	7.4 (3.8–14.7)	0.44 (0.36–0.54) *	40.1 (28.9–47.2) *
Normal	0.3 (0.3–0.4)	3.8 (3.5–4.3)	7.8 (2.9–12.7)	0.34 (0.24–0.43)	30.6 (26–36.2)
Daytime BP					
Abnormal	0.4 (0.3–0.4)	4.2 (4–5) *	6 (3.2–13.8)	0.42 (0.36–0.54) *	39.1 (29.5–46.9) *
Normal	0.3 (0.3–0.4)	3.8 (3.4–4.3)	7.8 (3–13)	0.34 (0.24–0.43)	30.6 (26–35.2)
Nighttime BP					
Abnormal	0.4 (0.3–0.4)	4.2 (3.9–4.8) *	4.6 (3.2–12.9)	0.42 (0.33–0.53) *	37 (30.7–47.2) *
Normal	0.3 (0.3–0.4)	3.8 (3.4–4.2)	8.1 (2.8–13.2)	0.34 (0.23–0.42)	30.4 (25.8–35.1)
BP load					
Abnormal	0.4 (0.3–0.4)	4.1 (3.6–4.8) *	5 (3–11.7)	0.38 (0.24–0.46)	32.9 (29.2–40.7) *
Normal	0.3 (0.3–0.4)	3.8 (3.4–4.1)	8.7 (4–15.3)	0.34 (0.24–0.43)	29.7 (25.6–32.9)
Night dipping					
Abnormal	0.3 (0.3–0.4)	3.9 (3.4–4.3) *	6.5 (3.3–10.7)	0.37 (0.24–0.49)	31.1 (29.1–41) *
Normal	0.3 (0.3–0.4)	3.8 (3.6–4.5)	9.3 (2.5–15.7)	0.34 (0.24–0.42)	30.6 (25.9–36.8)
ABPM profile					
Abnormal	0.4 (0.3–0.4)	4 (3.6–4.6) *	7.2 (3.3–11.7)	0.39 (0.24–0.46)	31.8 (28.1–40) *
Normal	0.3 (0.3–0.4)	3.8 (3.4–4.2)	9.3 (2.2–16.5)	0.31 (0.24–0.42)	28.8 (25.7–32.9)

Data are given as median (25th percentile–75th percentile). * *p* < 0.05 by the Mann-Whitney U-test. cIMT = carotid artery intima-media thickness. PWV = pulse wave velocity. FMD = flow-mediated dilatation. AASI = ABPM-derived arterial stiffness index. LVMI = left ventricular mass index.

**Table 5:** Adjusted regression model estimates (betas, *p*-values) of the association between BP load and NO-related biomarkers in children with CKD.

Dependent Valuable	Explanatory Valuable	Adjusted ^a^		Model	
		***Beta***	***p-*Value**	***r***	***p-*Value**
24-h systolic BP	Citrulline-to-arginine ratio	−0.288	0.006	0.613	<0.001
24-h diastolic BP	Citrulline-to-arginine ratio	−0.409	<0.001	0.625	<0.001
SBP load	Citrulline-to-arginine ratio	−0.331	0.001	0.691	<0.001
	Citrulline	−0.240	0.032		
DBP load	Citrulline-to-arginine ratio	−0.289	0.004	0.642	<0.001

^a^ Adjusted for age, sex, creatinine, and NO-related biomarkers.

**Table 6:** Correlation between NO-related biomarkers and cardiovascular surrogate markers in children with CKD stage G1–G3.

CV Markers	cIMT		PWV		FMD		AASI		LV Mass		LVMI	
	*r*	*p*	*r*	*p*	*r*	*p*	*r*	*p*	*r*	*p*	*r*	*p*
Citrulline	−0.028	0.813	0.035	0.771	0.027	0.835	0.092	0.443	−0.127	0.291	−0.143	0.233
Arginine	0.346	0.003 *	0.258	0.029 *	0.026	0.845	0.21	0.079	−0.197	0.099	0.21	0.078
ADMA	0.084	0.483	−0.074	0.537	−0.177	0.173	0.118	0.328	−0.003	0.978	−0.128	0.287
SDMA	−0.122	0.307	−0.051	0.673	0.076	0.561	0.039	0.745	0.079	0.515	−0.21	0.079
Arginine-to-ADMA	0.224	0.059	0.232	0.05	0.083	0.525	0.074	0.537	0.194	0.104	0.209	0.08
ADMA-to-SDMA	0.006	0.961	−0.009	0.942	−0.089	0.494	−0.009	0.942	−0.127	0.291	0.046	0.688
Citrulline-to-Arginine	−0.342	0.003 *	−0.159	0.181	0.001	0.991	−0.055	0.648	−0.267	0.024 *	−0.302	0.011 *

cIMT = carotid artery intima-media thickness; PWV = pulse wave velocity; FMD = flow-mediated dilatation; AASI = ABPM-derived arterial stiffness index; LV mass = Left ventricular mass; LVMI = Left ventricular mass index. * *p* < 0.05 by the Spearman’s correlation.
